# Supplementary material for: PoLoCo: a reproducible pooled low-coverage workflow for draft genome assembly and allele frequency analysis from ethanol-preserved small non-model invertebrates
Source: GigaByte. 2026 Oct 13;2026:gigabyte195. doi: 10.46471/gigabyte.195 (PMC13688668; doi:10.46471/gigabyte.195)
Supplement: Peer review history

# Reviewer report: Review MS


Reviewer name and names of any other individual's who aided in reviewerHailinDo you understand and agree to our policy of having open and named reviews, and having your review included with the published manuscript. (If no, please inform the editor that you cannot review this manuscript.)YesIs the language of sufficient quality?YesPlease add additional comments on language quality to clarify if neededIs there a clear statement of need explaining what problems the software is designed to solve and who the target audience is? YesAdditional CommentsIs the source code available, and has an appropriate Open Source Initiative license <a href="https://opensource.org/licenses" target="_blank">(https://opensource.org/licenses)</a> been assigned to the code?YesAdditional CommentsAs Open Source Software are there guidelines on how to contribute, report issues or seek support on the code?YesAdditional CommentsIs the code executable?YesAdditional CommentsIs installation/deployment sufficiently outlined in the paper and documentation, and does it proceed as outlined?YesAdditional CommentsIs the documentation provided clear and user friendly?YesAdditional CommentsAdditional CommentsIs there a clearly-stated list of dependencies, and is the core functionality of the software documented to a satisfactory level?YesAdditional CommentsHave any claims of performance been sufficiently tested and compared to other commonly-used packages? NoAdditional CommentsAdditional CommentsAre there (ideally real world) examples demonstrating use of the software? YesAdditional CommentsAdditional CommentsAny Additional Overall Comments to the AuthorThis study presents PoLoCo, an integrated and reproducible workflow for draft genome assembly and Pool-seq allele frequency analysis using ethanol-preserved small invertebrates. The authors completed the genome assembly of Entomobrya nivalisand and the population genomic analysis based on degraded pooled samples. The research topic is practical, and the pipeline is fully open-source, which fits the journal scope. However, several key issues regarding rationality of experimental design, result interpretation and discussion depth need to be addressed thoroughly before accepted. 1.Recent advances in museomics and short-read assembly for ethanol-preserved specimens are insufficiently reviewed. Please clarify the core innovations of PoLoCo compared with existing pipelines. 2.The source and impact of the 85.8% ANI difference between the assembled draft genome and the published reference genome are not adequately explained. This divergence is substantially higher than the expected nucleotide similarity within conspecific individuals. Please explicitly distinguish between contributions from geographic population genetic differentiation, ethanol preservation-induced sequencing artifacts, and inherent errors of short-read assembly. Additionally, supplement analyses to verify whether this difference introduces systematic biases in subsequent SNP calling and allele frequency estimation. 3.The selection of key filtering thresholds lacks justification and robustness validation. The criteria of "coverage in at least 55 out of 82 populations" and "200bp distance-based thinning" are adopted without describing the decision-making process. No tests were conducted to evaluate how alternative thresholds (e.g., 40/82 populations, 100 bp/500 bp thinning) would affect the size of the final SNP dataset and the outcomes of population genetic inferences, undermining confidence in the stability of the results. 4.Reference bias analysis is limited to superficial SNP characteristics and does not extend to downstream biological conclusions. The current comparison only addresses SNP count, allele frequency distribution, and Ts/Tv ratio. Please add parallel population genomic analyses using both the draft genome and the NCBI reference, including PCA clustering and pairwise FST correlation, to quantify how reference choice influences core biological interpretations of population structure and differentiation. 5.The limitations and applicable scope of the PoLoCo workflow are not clearly defined. The manuscript does not specify the minimum DNA quality requirements (e.g., fragment length distribution, concentration threshold) for successful implementation. It also fails to discuss potential challenges and parameter adjustments when processing museum specimens preserved for longer periods (e.g., >10 years) or pooled samples with different numbers of individuals. 6.Critical tables suffer from severe formatting issues and incomplete data. Table 1 has misaligned columns and incorrectly grouped workflow steps, while Table 2 is missing core assembly metrics (e.g., total contig count, N50, L50) for side-by-side comparison with the published reference. Table 4 contains empty cells in the "Handles degraded/preserved DNA" column for the assessPool pipeline. All tables must be restructured and completed to meet the journal's data presentation standards.RecommendationMajor Revisions


# Reviewer report: Review MS


Reviewer name and names of any other individual's who aided in reviewerYing SUNDo you understand and agree to our policy of having open and named reviews, and having your review included with the published manuscript. (If no, please inform the editor that you cannot review this manuscript.)YesIs the language of sufficient quality?YesPlease add additional comments on language quality to clarify if neededIs there a clear statement of need explaining what problems the software is designed to solve and who the target audience is? YesAdditional CommentsThe introduction clearly identifies the current lack of reference genomes for small invertebrates, such as specimens routinely preserved in ethanol, due to DNA degradation, and explains how this shortage hinders population genomic analyses using Pool-seq. The intended audience is explicitly researchers who work with non-model organisms and degraded DNA samples.Is the source code available, and has an appropriate Open Source Initiative license <a href="https://opensource.org/licenses" target="_blank">(https://opensource.org/licenses)</a> been assigned to the code?YesAdditional CommentsThe source code has been made available via Zenodo and GitHub, and is explicitly released under the MIT License.As Open Source Software are there guidelines on how to contribute, report issues or seek support on the code?YesAdditional CommentsThe article provides a link to the GitHub repository, offering users a standard open-source community channel for reporting issues and seeking support.Is the code executable?Unable to testAdditional CommentsThe installation instructions provided in the github repository are clear, and the setup process completed successfully on my side. However, the current workflow relies on creating multiple conda environments. To streamline deployment and reduce overhead, I recommend consolidating these into a single, unified environment file, assuming dependency conflicts permit. Unfortunately, the raw sequencing reads linked via the ENA accession number provided in the manuscript are currently inaccessible (an 'Record not found' error). Consequently, I was unable to perform a full, end-to-end stress test of the pipeline using the authors' data. While PoLoCo is presented as a reproducible end-to-end pipeline, its current architectural logic does not guarantee successful execution. My primary concern is the structural flow: the default launcher executes steps 1 through 8 in a fixed linear sequence. However, the mapping and pool-seq modules (steps 2 and 4) require a draft reference genome located at ref/poloco_draft.fa, whereas the de novo assembly module (step 6) is executed later in the process. Furthermore, the output of step 6 is not automatically directed or symlinked back to that reference directory. Thus, a run-all command implicitly assumes a pre-existing reference, leaving the assembly and downstream pool-seq tracks logically disconnected. As written, a complete run requires substantial manual intervention, such as manual reference placement, step reordering, and re-running the mapping module post-assembly. Moreover, upon reviewing the codebase, several internal inconsistencies weaken the claim of broad reproducibility. First, step 1 calls a qc script that expects a summary file which the pipeline itself never generates. Second, step 4 utilizes hard-coded filtering parameters, such as requiring a minimum representation in exactly 55 pools. These parameters are explicitly tied to the authors' specific study design rather than being exposed as user-defined variables, heavily limiting the tool's generalizability for other datasets. Furthermore, the optional annotation module in step 8 relies on oversimplified tool chaining that may fail to parse or match the actual formatting of standard annotation tool outputs. Finally, input validation within the scripts appears limited. Missing files or incompatible sample naming conventions may not trigger an error until late in the workflow, potentially wasting significant computational time. Ultimately, the repository reads more like a collection of study-specific custom scripts rather than a logically closed, generalized pipeline capable of autonomous, self-consistent execution.Is installation/deployment sufficiently outlined in the paper and documentation, and does it proceed as outlined?YesAdditional CommentsThe manuscript describes the implementation of modular conda environments and slurm scripts, thereby ensuring compatibility with standard computing cluster infrastructures.Is the documentation provided clear and user friendly?YesAdditional CommentsIs there enough clear information in the documentation to install, run and test this tool, including information on where to seek help if required?YesAdditional CommentsProcessing steps are scripted in the repositories (zenodo and github), which also offers optional modules for assembly validation and downstream annotation tasks.Is there a clearly-stated list of dependencies, and is the core functionality of the software documented to a satisfactory level?YesAdditional CommentsThe manuscript lists the core dependencies, including fastp, megahit, bwa-mem, samtools, and PoPoolation2, along with their specific versions.Have any claims of performance been sufficiently tested and compared to other commonly-used packages? YesAdditional CommentsTable 1 provides detailed benchmarks for runtime, cpu usage, and memory consumption. Table 4 presents a clear feature comparison between PoLoCo and existing Pool-seq pipelines, such as PoPoolation2 and ANGSD.Is test data available, either included with the submission or openly available via cited third party sources (e.g. accession numbers, data DOIs)?NoAdditional CommentsDraft genomes and associated reports can be found in figshare. However, although the manuscript states that the raw Illumina sequencing data were deposited in the ENA (PRJEB111482), attempting to access the provided link (https://www.ebi.ac.uk/ena/browser/view/PRJEB111482) results in an 'Record not found' error.Are there (ideally real world) examples demonstrating use of the software? YesAdditional CommentsThe study conducted a full application demonstration using 82 real-world Entomobrya nivalispooled samples collected from the 135 plots located in the ConFoBi Forest.Is automated testing used or are there manual steps described so that the functionality of the software can be verified?NoAdditional CommentsPoLoCo relies on manual, standards‑based verification rather than automated testing. Its functionality can be confirmed by reproducing the published case study using the provided conda environments, slurm scripts, and public datasets.Any Additional Overall Comments to the AuthorWhile PoLoCo provides a much-needed, reproducible framework for handling degraded invertebrate samples, I strongly encourage the authors to consider the following technical adjustments to mitigate potential alignment biases and improve the workflow's adaptability: (1) Risk of alignment bias exists for highly fragmented assemblies. The draft genome generated in this study exhibits extreme fragmentation, with an N50 of only 5,974 bp across 142,936 contigs. When using bwa-mem for short-read alignment, highly fragmented genomes and potential repetitive regions create a high risk of reads being erroneously mapped to non-homologous contigs. I recommend that the authors explicitly detail the logic behind the mapping quality thresholding in the filtering steps. Additionally, the discussion should address whether competitive mapping strategies could be introduced to mitigate false-positive snp discovery arising from assembly fragmentation. (2) Although the assembly achieved a high BUSCO completeness of 97.7%, the high heterozygosity of pooled samples and DNA degradation may still compromise allele frequency estimation for certain functional genes. I suggest adding a paragraph to the Conclusion section (probably conclusion & limitation) to address how assembly contiguity, specifically the fragmentation of functional genes across multiple contigs, might limit downstream functional enrichment analyses or the ecological interpretation of allele frequency shifts. (3) The current pipeline retains a locus only if it is covered in at least 55 of the 82 pooled populations. Could the authors clarify how this specific threshold was chosen and what the biological or statistical rationale is for requiring ~67% representation? Because a fixed, experience-based cutoff might limit the workflow's adaptability for users with vastly different sample sizes or coverage distributions, it would be helpful to explicitly explain this parameter choice. Have the authors also considered offering an automated, data-driven threshold-setting option to make the pipeline more robust for varying project scales? (4) The manuscript notes that PoLoCo is suitable for landscape genomics. To enhance the workflow's utility for ecology and evolutionary research, I strongly encourage the authors to include optional downstream modules that calculate population genetic differentiation indices and implement permutation tests. Such tests are critical for evaluating allele frequency differences among non-independent populations.RecommendationMajor Revisions


# Reviewer report: Re-Review MS


Indicate in the comments box below whether you are happy with the changes made or if the manuscript is unacceptable.Comments on revised manuscriptThe authors have provided thorough responses to all raised concerns. I have no further comments and recommend acceptance of the manuscript.


# Reviewer report: Re-Review MS


Indicate in the comments box below whether you are happy with the changes made or if the manuscript is unacceptable.Comments on revised manuscriptMinor revision. The authors provided an exceptionally thorough and honest revision. Rather than addressing my concerns at the level of manuscript wording, they re-engineered the PoLoCo repository itself and, importantly, disclosed and corrected errors. This level of transparency is commendable and exactly what a technical release in GigaByte should exemplify. I have re-examined the revised repository and data resources: (1) The ENA BioProject PRJEB111482 now resolves and lists 83 runs matching the manuscript. (2) The Zenodo archive resolves. (3) All claimed new components are present in the repository, including scripts/00_check_inputs.sh, scripts/02_assembly.sh (automatic reference preparation/indexing), scripts/03_validation.sh (MUMmer4/dnadiff), reordered run_poloco_pipeline.sh, hpc/submit_poloco_slurm.sh + run_poloco_slurm_step.sh, configs/install_poloco_conda_envs.sh, configs/poloco_config.sh + user-config template + smoke-test config, smoke_test/data/, ena_example/ manifest and downloader, and scripts/08_neutral_connectivity.sh with example coordinates. The unvalidated 08_annotation.sh has been removed as stated. Point-by-point evaluation of the responses to my previous comments (1) Retaining modular environments while providing a single installer (install_poloco_conda_envs.sh) is a sensible compromise given the conflicting toolchains; the rationale is convincing. (2) The BioProject is now public, and the added manifest + resumable, checksum-verified downloader goes beyond what I asked for. (3) The Step 0-8 reordering, automatic copying/indexing of the assembly to ref/poloco_draft.fa, and afterok-based SLURM submission make the workflow logically closed. Figure 1 now correctly reflects the execution order. (4) The preprocessing step now writes fastp_summary.tsv consumed by the QC scripts, plus an integrated QC workbook. (5) Rationale for the 55/82 threshold is now explained as a pragmatic case-study choice, with configurable absolute/proportional modes. (6) Fragile annotation module was removed. (7) Late input validation has been ddressed with the new step 0 check in both execution routes. (8) The revised code now constitutes a genuine generalized pipeline. Overall scientific comments: All of my previous concerns have been addressed satisfactorily. (1) MAPQ=30/BASEQ=20 are now documented and configurable; the overstated claims ("fragmentation does not substantially affect SNP detection", "strong collinearity", "less reference bias") have been removed or moderated; competitive/multi-reference mapping is discussed as future work; and the corrected N50 is honestly reported. (2) Functional-gene limitation paragraph has been added. (3) The new optional Step 8 (PCA, genetic/geographic distances, Mantel test with 9,999 permutations, demonstrated on the full 82 population matrix) adequately addresses my request while sensibly keeping scope limited. Remaining minor comments on the revised text: (1) Line 498-499 cites reference [48] (Zhang et al., "Applications of transformer-based language models in bioinformatics") to support "current recommendations for transparent and reproducible genomic workflows". This reference is about language models and is unrelated to workflow reproducibility. Please replace it with an appropriate citation (e.g., a reproducible-workflow/FAIR-software guideline paper). (2) Lines 326-327 state that "GC content distributions (Figure 2d) were consistent and showed most pools clustered 354 around 37.0 to 38.9%. Only one pool was an outlier and was considerably lower at 32.0% GC". However, Figure 2d shows a substantial right tail, roughly 20 pools with GC between ~40% and ~58%. Similarly, lines 322–324 describe mapping rates as "generally high", while Figure 2c shows about ten pools with mapping rates below ~50%. Please reconcile the text with the figures, report the actual mapping-rate range, and briefly comment on whether the elevated-GC / low-mapping pools may reflect residual exogenous reads, which is a plausible occurrence for whole-body extractions of ethanol-preserved specimens and whether this motivated the retention filters. (3) Line 223 states 55/82 (67%), while line 230 gives "a proportional threshold of 0.65 for the present 82-pool case study" and the response letter uses ~65% and ~67% in different places. 55/82 = 67.1%; note that 0.65 × 82 = 53.3 pools, which would not reproduce the case-study setting. Please use one consistent value (0.67) throughout. (4) Line 276-277, "usable data retention was good, with 94.2% of the raw reads filtered". Should "filtered" be "retained"? (5) As to Table 2, (a) Please state what "Misassemblies = 12" means for the NCBI reference column (misassemblies are defined relative to a reference; the reference compared against itself should be 0; presumably this is reciprocal QUAST output, but it needs a footnote). (b) "Largest contig > 1 Mb" should give the exact value. (c) Since aligned-region identity is symmetric (87.96% in both columns), consider a footnote to prevent misreading it as two independent measurements. (6) The 46.3% duplicated BUSCOs and the 411 Mb assembly size (vs. 301 Mb for the reference) suggest substantial uncollapsed heterozygosity from pooled input. A brief note that haplotig purging could reduce redundancy in future applications or why it was not applied would be useful to readers.


# Editor report: Accept


Editor’s AssessmentThe manuscript is ready for formal acceptance.Editor’s AssessmentThe manuscript is ready for formal acceptance.
